# School eye health in Nepal: A holistic model

**Published:** 2017

**Authors:** Sanjay Kumar Singh, Sudhir Thakur, Afaque Anwar

**Affiliations:** Programme Director, Eastern Regional Eye Care Programme, Biratnagar, Nepal; Programme Coordinator, Eastern Regional Eye Care Programme, Biratnagar, Nepal; Health Educator, Biratnagar Eye Hospital, Biratnagar, Nepal

**Figure:**
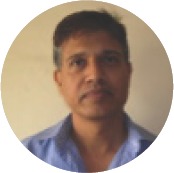
Dr Sanjay Kumar Singh

**Figure:**
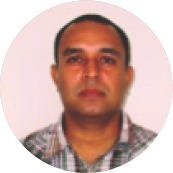
Sudhir Thakur

**Figure:**
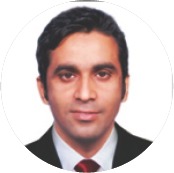
Afaque Anwar

**The integration of eye health into comprehensive school eye health programmes not only helps to identify children with significant refractive error and associated ocular morbidities but also helps to promote a healthy school environment.**

**Figure:**
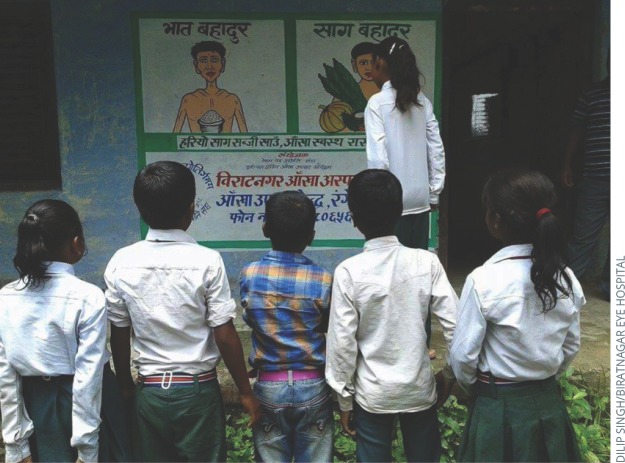
Sensitising school children on eye health through wall painting of *saag-bahadur bhaat-bahadur.* NEPAL

Health including visual health is inextricably linked to school achievement, quality of life and economic productivity.^1^ The school eye health programme organised by eastern regional eye care programme (EREC-P) in the eastern region of Nepal is designed to improve the visual health of pupils, by directly influencing school personnel, families and indirectly influencing other members of the community through schools.

Globally, 19 million children live with visual impairment, and approximately 12 million children have significant refractive error.^2^ The prevalence of refractive error (0.5 dioptres or more for myopia, 1.00 dioptre or more for hypermetropia and ≥ 0.75 DC for astigmatism) among school going children in eastern part of Nepal was 8.6% and myopia was the most common type (44.79%) of refractive error.^3^ As per the recently adopted National Eye Health Policy 2017 the school eye health programme needs to develop and strengthen comprehensive eye examination by eye health professionals at the time of school enrollment in Nepal.

The integration of eye health into comprehensive school health programmes not only helps to identify children with significant refractive error and associated ocular morbidities but also helps to promote a healthy school environment. With this, eye health education can reach a large number of children and their families through a child-to-child approach.^4^

The elements of the school eye health programme are as follows:

1. Primary eye care for early diagnosis and treatment of common eye diseases, management of refractive errors and low vision by providing high quality, appealing and free of cost spectacles and low vision devices.
2. Increasing awareness about healthy school environments amongst children, teachers and communities.
3. Making schools inclusive for children with visual impairment so that they can learn together with normal children.

Activities include:

- Eye care teams screen students and staff, provide medicine and spectacles at schools, and refer children with complex refractive error and associated ocular morbidities to a base hospital or eye care centre for further evaluation by an ophthalmologist.
- Health promotion and prevention activities on a sustained basis through art by conducting school eye health exhibitions and wall painting.
- Counselling students who received spectacles along with their families and school authorities to ensure they wear spectacles at school and at homes.
- Promoting inclusive education for children with disabilities through advocacy for improved accessibility such as ramp, classroom setting for children with visual impairment especially low vision, etc., and also sensitise children, adults and teachers on how to help and interact with children with visual impairment.

## Established models of school screening programme in Nepal

### 1. Teacher-oriented approach

A single day's training was provided to school teachers to identify, refer and enable children with complex refractive errors and associated ocular morbidities for vision screening at a nearby primary eye care centre or base hospital, by giving a referral slip. The referred children had to go through a comprehensive eye examination to receive medicine, spectacles and low vision devices free of cost. This approach uses local resources and these resources are based on the willingness of trained teachers and support from educational authorities. It requires accuracy of screening in school settings. The main drawback of the programme was the high referral dropout.

### 2. Eye care team approach

School screenings were done by an eye care team comprising of an optometrist or ophthalmic assistant (OA) and an eye health worker. Eye health workers were responsible for conducting vision screening whereas optometrist or ophthalmic assistants did the screening and retinoscopy of all children irrespective of visual acuity. Referral to an ophthalmologist was done only for children whose best corrected visual acuity (BCVA) in their better eye was less than or equal to 6/9 and had associated ocular morbidities that warrant further evaluation by an ophthalmologist. The instruments used during the screening programme were Snellen chart, ophthalmoscope, retinoscope and a torch light. Even though this approach enhances the accuracy of the programme, questions about tracking children referred to a hospital or eye care centre, and prevention and promotion aspects still remain.

### 3. Holistic approach

This approach involved eye screening, health education and inclusion.

## School Screening

**Figure 1:**
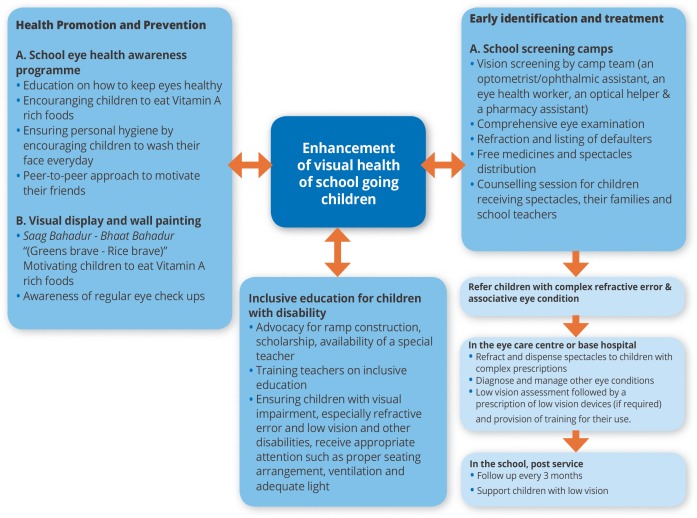
Holistic approach of school screening camp

Key learnings from the above two approaches led us to reformulate the screening programme using a mixed approach (Figure 1). Screening is done by camp team comprising an optometrist or ophthalmic assistant (OA), eye health worker, optical helper and a pharmacy assistant.

- Eye health worker is responsible for conducting vision screening and eye health exhibitions,
- Optometrist or ophthalmic assistant for screening and retinoscopy of all children irrespective of visual acuity,
- Optical helper for processing and fitting of spectacles and
- Pharmacy assistant for medicine distribution.

The vision screening is followed by comprehensive eye examination, refraction and provision of free medicines and spectacles, provided as per the need at a school site and referring children with complex refractive errors and associated eye conditions to an ophthalmologist. A counselling session was organised for children who obtained spectacles along with their families and school authorities to ensure spectacle wearing in schools and homes. The session also addressed proper seating arrangement in classrooms to ensure attention and participation in class activities.

**Figure:**
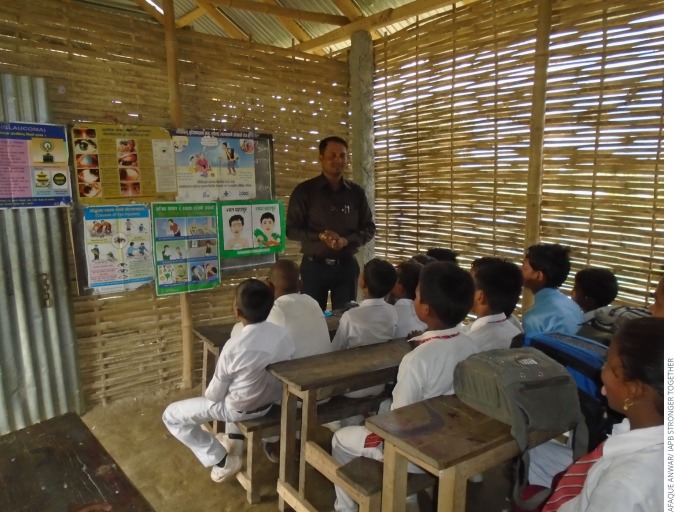
A Vitamin A Awareness Programme. NEPAL

## School eye health education

Along with screening, the school eye health awareness programme also included awareness about keeping eyes healthy by eating Vitamin A rich foods every day; attention to personal hygiene by making sure children wash their face daily and encouraging mass awareness through a child-to-child approach. A wall painting *“Saag Bahadur- Bhaat Bahadur”* (Motivating children to eat Vitamin ‘A’ rich foods daily) was also displayed at a school site to create awareness regarding eye health.

## Inclusion

Another unique aspect of the programme, were the activities to promote inclusive education in schools where children with disabilities study. These were advocacy for construction of ramps in schools and ensuring scholarships to children with disabilities. Sensitising important stakeholders in the government for mobilisation of funds to those children as well as providing orientation and training to teachers and school management on inclusive education was also part of this project. The aim of these activities was to sensitise and impart knowledge on inclusive education and create a welcoming learning environment in classrooms which will help in minimising the dropout rates of children with visual impairment.

Though this holistic approach had many positive outcomes one important concern was the long term follow up mechanism. Follow up of children with refractive error and complex eye conditions by field level staffs every three months at schools, will ensure spectacle wearing and minimise the dropouts. This model holds promise in ensuring no child is left behind.

Key factors that contribute to success and sustainability of school eye health programme includes

- Continuous support and engagement of local education authorities
- Active participation of parents and care-takers
- Financial and technical support from public and private agencies
- Continuous commitment of the eye health workforce
- Continuous supply of high quality spectacles and low vision devices

Eye health is an essential part of any school health programme, should be aligned in such a way that services are available and accessible to all children. The school eye health programme should not only focus on correction of refractive errors, but also be comprehensive and holistic in nature.
